# ReMIND: The Brain Resection Multimodal Imaging Database

**DOI:** 10.1101/2023.09.14.23295596

**Published:** 2023-09-15

**Authors:** Parikshit Juvekar, Reuben Dorent, Fryderyk Kögl, Erickson Torio, Colton Barr, Laura Rigolo, Colin Galvin, Nick Jowkar, Anees Kazi, Nazim Haouchine, Harneet Cheema, Nassir Navab, Steve Pieper, William M. Wells, Wenya Linda Bi, Alexandra Golby, Sarah Frisken, Tina Kapur

**Affiliations:** 1 Brigham and Women’s Hospital, Harvard Medical School, Boston, MA, USA; 2 Computer Aided Medical Procedures, Technische Universität München, Munich, Germany; 3 Department of Health Science, University of Ottawa, Ottawa, Canada; 4 Computer Science and Artificial Intelligence Laboratory, Massachusetts Institute of Technology, Boston, USA

## Abstract

The standard of care for brain tumors is maximal safe surgical resection as the first step. Neuronavigation augments the surgeon’s ability to achieve this but loses validity due to brain shift as surgery progresses. Moreover, many gliomas are difficult to distinguish from adjacent healthy brain tissue. Intraoperative MRI (iMRI) is a useful surgical adjunct that can be used to visualize the residual tumor and brain shift. Intraoperative ultrasound (iUS) serves a similar purpose, while also being faster and easier to incorporate into the workflow. However, it provides lower contrast between tumor tissue and normal brain tissue as compared to intraoperative MRI. With the success of data-hungry Artificial Intelligence (AI)/Machine Learning (ML) algorithms in advancing the state of the art in medical image analysis, the benefits of sharing well-curated data can not be overstated. To this end, we provide here the largest publicly-available MRI and intraoperative ultrasound imaging database of surgically treated brain tumors, including gliomas (n=92), metastases (n=11), and others (n=11). This collection contains 369 preoperative MRI series, 320 3D intraoperative ultrasound series, 301 intraoperative MRI series, and 356 segmentations collected from 114 consecutive patients at a single institution. We expect this data to be a resource for computational research in brain shift and image analysis as well as for neurosurgical training in the interpretation of intraoperative ultrasound and iMRI.

## Background & Summary

Image guidance with computerized navigation based on preoperative MRI was introduced as a surgical adjunct in the 1990s. It facilitates greater resection accuracy by helping the surgeon plan the approach and locate the boundaries of the intended resection. Neuronavigation loses validity as the surgery progresses due to non-linear deformations of the resection cavity and brain shift^1–5^. Intraoperative imaging such as MRI and ultrasound serve to alleviate this issue. While iMRI provides high-contrast images, they can take several minutes up to an hour to acquire. On the other hand, iUS provides relatively lower-contrast images, but they can be acquired within minutes.

**Figure 1.**
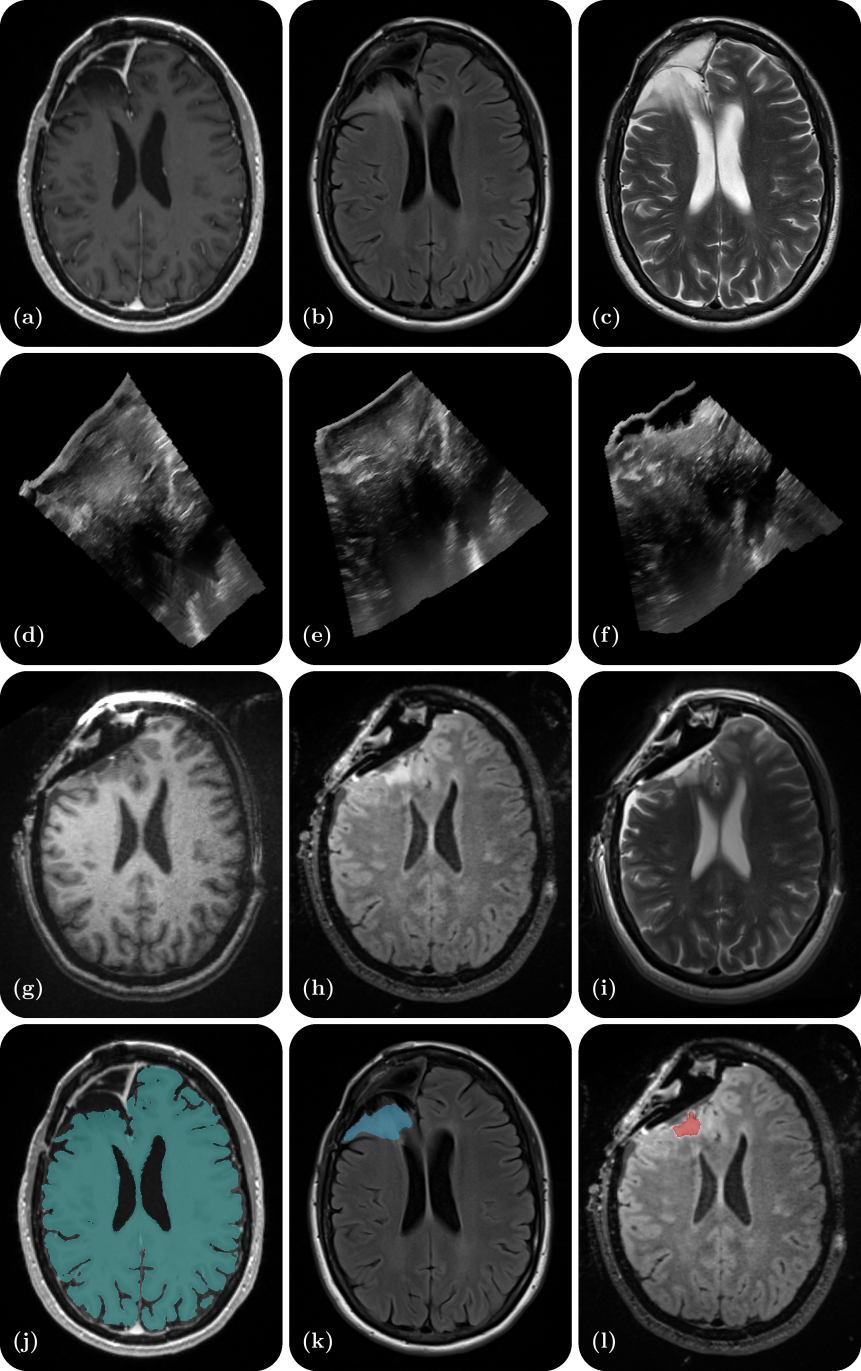
Illustrative example of one dataset - a right frontal lobe recurrent WHO Grade II Oligodendroglioma (IDH-positive, 1p/19q co-deleted). (a) Preoperative contrast-enhanced T1-weighted MR; (b) Preoperative T2-weighted MR; (c) Preoperative T2 FLAIR MR; (d) Intraoperative US prior to dural opening; (e) Intraoperative US post dural opening; (f) Intraoperative US prior to iMRI; (g) Intraoperative contrast-enhanced T1-weighted MRI; (h) Intraoperative T2 FLAIR MRI; (i) Intraoperative T2-weighted MRI (BLADE); (j) Cerebrum segmentation on the preoperative contrast-enhanced T1-weighted MRI; (k) Tumor segmentation on the preoperative T2 FLAIR MRI; (l) Residual tumor segmentation on the intraoperative T2 FLAIR MRI.

Research in the field of medical imaging and image-guided therapy is increasingly seeking large datasets to develop and test AI/ML based algorithms. However, sharing patient datasets for public research use is challenging due to the significant resources required for data curation as well as the need to ensure patient privacy. Moreover, datasets that combine multimodal imaging from both preoperative and intraoperative acquisitions in the same patient are particularly scarce. To address this gap, we have curated a database from neurosurgical procedures conducted in the Advanced Multimodality Image Guided Operating (AMIGO) suite at the Brigham and Women’s Hospital. This database represents the largest publicly-available collection of preoperative MRI, intraoperative MRI (iMRI), and intraoperative ultrasound (iUS) data from surgically treated brain tumors. It contains 92 gliomas, 11 metastases, and 11 non-glioma pathologies. The dataset includes 369 preoperative MRI series, 320 three-dimensional iUS sweeps, 301 iMRI series, and 356 segmentations obtained from 114 consecutive patients who underwent image-guided resection at a single institution. Additionally, each case contains segmentations of the preoperative tumor, the pre-resection cerebrum, and the previous resection cavity derived from the preoperative MRI (if applicable), as well as any residual tumor identified on the iMRI. For reference, Figure 1 provides an illustrative example of the contents of each dataset and all available segmentations.

With this work, we build upon the effort initiated by the Montreal Neurosurgical Institute (MNI) and St. Olav University Hospital (Trondheim, Norway) to publicly share MRI and iUS images acquired for brain tumor patients through the BITE^6–8^ (Brain Images of Tumors for Evaluation) and RESECT^9,10^ (REtroSpective Evaluation of Cerebral Tumors) databases (Table 1) respectively.

## Methods

This section describes all the procedures followed to acquire and curate the data within the ReMIND collection, including experimental design, data acquisition, data annotation, and computational processing (e.g., format conversion, defacing).

### Patient cohort

The ReMIND database comprises 123 consecutive patients who were surgically treated with image-guided tumor resection in the AMIGO Suite at the Brigham and Women’s Hospital (Boston, USA), between November 2018 and August 2022, using both intraoperative ultrasound (iUS) and intraoperative MRI (iMRI). Of the 123 cases, 9 were excluded due to corrupted or poor-quality data, resulting in a final cohort of 114 cases. Summary demographics can be found in Table 2.

The patients in the study were treated according to the current standard of care. Collection, analysis, and release of the ReMIND database has been performed in compliance with all relevant ethical regulations. The Institutional Review Board at the Brigham and Women’s Hospital approved the protocol, and informed consent was obtained from all participants.

### Surgical Setup with Neuronavigation

Intraoperative neuronavigation was performed using the optical tracking version of the ‘Curve’ Dual Display system (Brainlab AG, Munich, Germany). The patient reference frame was established by rigidly securing the MRI-safe ‘Standard Cranial Reference Array with 4 Marker Spheres’ (Brainlab AG, Munich, Germany) to the MRI-safe IMRIS head holder (IMRIS Inc., Minnesota, USA). The precalibrated ‘Softouch Pointer’ (Brainlab AG, Munich, Germany) was then used to perform image-to-patient registration with the ‘Cranial Navigation’ module (Brainlab AG, Munich, Germany). The nasion, left lateral canthus, and right lateral canthus were located on the patient using the tracked pointer to establish an initial registration. This was followed by the acquisition of a dense set of points on the skin over bony prominences to refine the patient registration to the skin surface on preoperative imaging. The quality of the registration was visually verified by the surgeon and improved when necessary by collecting additional registration points.

### Clinical, Demographic, and Pathology data

Demographic information, including age, sex, and ethnicity, was obtained from the corresponding patient medical records. The age range of the included population was 20–76. The ratio of male:female was equal to 61:53. Moreover, clinico-pathologic data such as the tumor type, tumor grade, radiological characteristics upon contrast administration, tumor location, and the reoperation status were assessed by the treating neurosurgeons. Tumor type and grade were specified according to the World Health Organization (WHO) 2021 Classification of Tumors of the Central Nervous System.^11–14^. Additionally, tumors were classified into one of 3 categories based on proximity to the functional cortex (non-eloquent, near eloquent, and eloquent).

Table 2 shows a summary of the clinical metadata. A number of the surgeries were reoperations (44.7%) and a minority were performed awake (4.4%). There was an equal number of right- and left-sided craniotomies. The majority of the patients treated were classified as ‘gliomas, glioneuronal, and neuronal tumors’ (83.3%) and were primarily Astrocytomas (34.7%). IDH-mutations, 1p/19q-codeletions, and MGMT promoter methylation status were reported in 100%, 67.5%, and 94.3% of the adult-type diffuse gliomas, respectively. The majority of the ‘gliomas, glioneuronal, and neuronal tumors’ were CNS WHO Grade 4 (38.9%). (67.5%) were located in eloquent areas based on the fMRI, anatomic substrate, and neurophysiological monitoring. The full metadata table can be downloaded from our repository on The Cancer Imaging Archive (TCIA).

### Imaging data

The ReMIND database corresponds to a collection of preoperative MRI, intraoperative MRI (iMRI), and intraoperative ultrasounds (iUS). A summary of the acquisition parameters for preoperative and intraoperative MRI is provided in Table 1. The imaging data can be found and downloaded from our TCIA repository as well.

#### Preoperative MRI

Preoperative MRI comprises four structural MRI sequences: native T1-weighted (T1), contrast-enhanced T1-weighted (ceT1), native T2-weighted (T2), and T2-weighted fluid-attenuated inversion recovery (T2-FLAIR). These scans were acquired before surgery using various scanners at multiple institutions, making their acquisition parameters heterogeneous, as shown in Table 1. Most of the preoperative imaging was performed on a 3T (71.1%) MRI scanner with Siemens (87.7%) being the most common manufacturer.

#### Intraoperative MRI (iMRI)

Unlike preoperative MRI, all intraoperative MRI were acquired in the AMIGO suite at the Brigham and Women’s Hospital (Boston, USA) using a 3T wide-bore (70 cm) MRI scanner (Magnetom Verio, Siemens Healthineers, Erlangen, Germany) with an 8-channel head coil to evaluate the presence of residual targeted tissue once most or all of the targeted tissue was removed. Before the acquisition, a temporary closure of the craniotomy was performed. The entire acquisition process required 1–1.5 hrs. and included MRI safety procedures, instrument counts, preparation for scanning, and redraping to resume surgery after iMRI.

#### Intraoperative Ultrasound (iUS)

All iUS series were acquired using a sterilizable 2D neuro-cranial curvilinear transducer on a cart-based ultrasound system (N13C5, BK5000, GE Healthcare, Peabody, MA, USA) in the AMIGO suite. The ultrasound probe had a contact area of 29mm × 10mm and a frequency range of 5–13 MHz. The ‘Ultrasound Navigation Adapter Array’ together with the ‘Ultrasound Navigation Adapter Base - BK N13C5’ (Brainlab AG, Munich, Germany) were attached to the iUS probe to enable the Curve platform to track the probe relative to the patient. The imaging plane was chosen to be as parallel as possible to one of the three cardinal axes of the head (axial, sagittal, coronal). However, this was often limited by the size and shape of the craniotomy. The transducer was swept unidirectionally at a slow, consistent speed through the craniotomy. This specific motion, in conjunction with the tracking, enabled the reconstruction of a 3D volume from the tracked 2D sweeps using the ‘Ultrasound’ module within the ‘Elements’ software platform on the ‘Curve’ hardware system. Similar to the RESECT database^9^, 3D iUS acquisition was aimed to be performed at three distinct surgical time points:

1. **Pre-dura iUS:** between the craniotomy and the dural opening.
2. **Post-dura iUS:** after the dural opening but before any tumor resection was performed.
3. **Pre-iMRI iUS:** immediately before the iMRI was acquired, i.e. after substantial tumor resection was completed to the degree that either the surgeon was satisfied with the microscopically-visible extent of resection or that iMRI was needed to identify the remaining portion of the tumor.

When more than one iUS volume was acquired at a specific surgical time point, the acquisition with the best image quality, field of view, and maximal tumor coverage was included in this collection. In 22 cases, iUS acquisitions were unavailable at some surgical time points due to surgeon preference (15/22) or durotomies during craniotomies or previous surgeries (7/22). Such cases have an explanation detailed in the clinical metadata table available on TCIA.

### Segmentation data

Various segmentations were created to assist the surgical resection. These typically include manual segmentations of the preoperative whole tumor, preoperative tumor target (i.e., the radiologically identifiable tumor specifically targeted for resection), resection cavity resulting from prior surgery (i.e. in case of reoperation), intraoperative residual tumor, and the automatic segmentations of cerebrum and ventricles using the ‘Object Manipulation’ module (Brainlab AG, Munich, Germany). Only structures deemed necessary for the surgical resection by the attending neurosurgeon were segmented. Specifically, manual preoperative whole tumor segmentations are provided for 113 cases; preoperative tumor target segmentations are provided for 3 cases; manual previous resection cavity segmentations are provided for 21 cases; residual tumor segmentations are provided for 58 cases; and automated segmentations of the cerebrum and ventricles are respectively provided for 89 and 54 cases. All cerebrum, ventricle, and tumor segmentations were created preoperatively during the surgical planning stage. In contrast, residual tumor segmentations were created intraoperatively from iMRI.

### Data export

Preoperative MRI were accessed, selected, and co-registered using the ‘Image Fusion’ module within the ‘Elements’ software platform (Brainlab AG, Munich, Germany). After visual inspection, some MR series were excluded due to poor quality, the presence of artifacts, or a small field of view. Manual and automated segmentations were performed using the ‘Object Manipulation’ module. Intraoperative ultrasounds were tracked using the ‘Curve’ neuronavigation system, allowing them to be roughly registered with the preoperative images. Finally, intraoperative MRI were automatically registered with preoperative scans also using the ‘Image Fusion’ module in ‘Elements’. Note that image registration was performed by only updating image headers to avoid resampling errors. Images and segmentations were finally exported as NRRD files from the ‘Curve’ neuronavigation system using 3D Slicer via OpenIGTLink^15^.

### Data transmission, de-identification and format conversion

The de-identified imaging data and segmentation data were submitted to The Cancer Imaging Archive (TCIA) in DICOM format (imaging and segmentation) and NRRD format (segmentation). Experienced quality-control reviewers inspected images to ensure the data are fully de-identified and well-curated.

Data were fully de-identified by removing all health information identifiers and by applying a de-facing algorithm. Specifically, each MR scan was defaced by: 1/ affinely registering an MR template to the MR scan using NiftyReg^16^; 2/ applying the obtained affine transformation to the face mask of the template; 3/ applying the resampled face mask to the MR scan. The code of the algorithm is publicly available^1^. All the defaced scans were visually inspected, and 100% of them were successfully defaced.

The DICOM format is widely used in research as it is standardized, preserves all metadata (e.g. patient, session), and allows for long-term archival. For that reason, our imaging data was released in DICOM format on TCIA. Specifically, the preoperative and intraoperative MR images were converted from NRRD to DICOM series using 3D Slicer^17^. Moreover, the reconstructed 3D iUS images were converted to multi-frame DICOM using the dicom3tools software^18^. Finally, each segmentation was converted using DCMqi^19^ with the segmented MR DICOM data as the reference image.

### Data Records

All the imaging data and the metadata described here as the “ReMIND” collection are available as a publicly available repository of The Cancer Imaging Archive (TCIA) at: https://doi.org/10.7937/3rag-d070.

### Technical Validation

#### Image selection and registration steps

The quality of the preoperative MR images in this dataset and their co-registration were assessed by neurosurgeons during surgical planning. The quality of the image-to-patient registration step was visually verified by the surgeon and manually refined if needed.

#### Image de-facing

The derived de-facing masks for all cases were visually checked for quality. The developed tool was particularly robust: 100% of the preoperative and intraoperative MR scans were successfully defaced.

#### Imaging data conversion

To ensure that the data conversion step (from NRRD to DICOM) did not deteriorate raw imaging data, DICOM images were converted back to NRRD files using 3D Slicer. Then, the 3D image arrays of the original NRRD and the converted NRRD files were compared using the Simple Insight Toolkit^20^ (SimpleITK). The conversion process was found to preserve data information, i.e. the same values were found at each voxel location.

#### Segmentation data

Neurosurgeons assessed the quality of the manual segmentations in this dataset during surgical planning. Interobserver variability was measured for the manual tumor segmentations on 10 cases for further validation. Specifically, 5 preoperative T1-weighted MR series with gadolinium contrast and 5 preoperative T2-weighted MR (SPACE) series were annotated by two experts in fellowship positions with over 5 years of clinical experience in neuroradiology. We quantitatively measured the Dice score and average symmetric surface distance (ASSD) between the two sets of manual annotations. For manual tumor segmentation on post-contrast T1-weighted MRI, interobserver variability recorded a Dice score of 92.2% (SD 2.8%) and an ASSD score of 0.68mm (SD 0.34mm). For manual tumor segmentation on T2-weighted MRI, interobserver variability recorded a Dice score of 86.4% (SD 3.7%) and an ASSD score of 1.24mm (SD 0.22mm).

### Usage Notes

To view the imaging data, we recommend 3D Slicer^2^, which is a free and open-source platform for medical image informatics, image processing, and three-dimensional visualization. 3D Slicer supports the DICOM format. We also developed a custom 3D Slicer extension to create landmarks on images. The MRUSLandmarking extension is freely available in the 3D Slicer Extension Manager or at https://github.com/koegl/SlicerMRUSLandmarking.

## Figures and Tables

**Table 1.** Comparison against existing publicly available databases of preoperative and intraoperative imaging in brain cancer patients.

	MNI BITE^6–8^	RESECT^9, 10^	ReMIND
			
Group	Montreal	Trondheim	Boston
Publication Year(s)	2012^6^, 2014^7^, 2016^8^	2017^9^, 2022^10^	2023
Patients	14^7^ (+9^8^)	23	114
Tumor Type(s)	Low-Grade Gliomas High-Grade Gliomas	Low-Grade Gliomas	Low-Grade Gliomas High-Grade Gliomas Other tumors

Preoperative MRI	1.5T GE Signa EXCITE 3T Siemens Magnetom TIM Trio	1.5 Siemens Magnetom Avanto 3T Siemens Magnetom Skyra	Various 1.5T/3T/7T scanners (Siemens, GE, Hitachi)
Intraoperative MRI (iMRI)	- -	- -	3T Siemens Magnetom Verio 70 cm Wide-Bore
Postoperative MRI	1.5T GE Signa EXCITE	-	-
Smallest MRI Voxel Size	1 × 0.5 × 0.5 mm	1 × 1 × 1 mm	1 × 1 × 0.5 mm

Neuronavigation Processing Unit	IBIS NeuroNav	Sonowand Invite	Brainlab Curve Dual Display Brainlab Cranial Navigation, Brainlab Ultrasound
Neuronavigation Optical	Polaris	Polaris Spectra	Polaris Spectra for Brainlab
Tracking Unit	(Northern Digital Inc.)	(Northern Digital Inc.)	(Northern Digital Inc.)

Navigation Tracking Adapter for iUS Probe	TA003 (Traxtal Technologies Inc.)	Built-in	Adapter Base (41860-XX) Array (22595) (Brainlab AG)
iUS Processing Unit	HDI 5000 (Philips ATL)	Built-in	bk5000 (BK medical)
iUS Probe	4–7 Mhz Phased Array Transducer P7–4 (Philips ATL)	12–6 Mhz Flat Linear Array Transducer 12FLA-L (Sonowand AS)	13–5 Mhz Curved Array Array Transducer N13C5 (9062) (BK medical)
iUS Image Depth	6.5 cm or 8 cm	-	6.5 cm
iUS Reconstruction Resolution	2D: 0.2 × 0.2 mm 3D: 0.3 × 0.3 × 0.3 mm	0.14 × 0.14 × 0.14 mm to 0.24 × 0.24 × 0.24 mm	0.1 × 0.1 × 0.5 mm
Extradural iUS (before dural incision)	2D & 3D	3D	3D
Intradural iUS (after dural incision)	-	-	3D
Resection Control iUS	-	3D	-
Post-resection (pre-iMRI) iUS	2D & 3D	3D	3D

Segmentations	Preoperative Tumor (Manual)	Preoperative Tumor (Manual)^10^ Resection cavity in iUS (Manual)	Preoperative Tumor (Manual) Prior Resection Cavity (Manual) Preoperative Ventricles (Auto) Preoperative Cerebrum (Auto)
Landmarks	iUS to iUS post-resection iUS to MRI	iUS to iUS pre-resection iUS to MRI^9^	Not yet available

**Table 2.** Summary of the clinical metadata of the ReMIND data collection.

Clinical Data (N=114)			

**Demographics**		**Histopathology**	
Age	47.50 [32.75 – 59.00]	Gliomas, glioneuronal, and neuronal tumors	83.3% (95/114)
Sex		*Astrocytoma*	*34.7% (33/95)*
*Male*	*53.5% (61/114)*	*Glioblastoma*	*32.6% (31/95)*
*Female*	*46.5% (53/114)*	*Oligodendroglioma*	*25.3% (24/95)*
Race		*Other*	*7.4% (7/95)*
*White*	*90.4% (103/114)*	Meningiomas	0.9% (1/114)
*Asian*	*5.3% (6/114)*	Hematolymphoid tumors	0.9% (1/114)
*Declined*	*2.6% (3/114)*	Metastases	9.6% (11/114)
*Other*	*1.8% (2/114)*	Other	5.3% (6/114)
Ethnicity			
*Not Hispanic*	*94.7% (108/114)*	**Adult-type diffuse gliomas (n=88)**	
*Hispanic*	*4.4% (5/114)*		
*Declined*	*0.9% (1/114)*	**WHO grade**	
		*1*	*3.2% (3/88)*
**Preoperative MRI**		*2*	*31.6% (31/88)*
Tesla		*3*	*24.2% (23/88)*
*3T*	*71.1% (81/114)*	*4*	*38.9% (37/88)*
*1.5T*	*21.1% (24/114)*	*Not assigned*	*2.1% (2/88)*
*7T*	*1.8% (2/114)*		
*1.16T*	*1.8% (2/114)*	**Molecular genetics**	
Eloquent		IDH (n=88)	
*Yes*	*67.5% (77/114)*	*IDH-mutant*	*64.8% (57/88)*
*No*	*32.5% (37/114)*	*IDH-wildtype*	*35.8% (31/88)*
Enhancement	57.9% (66/114)	1p/19q (n=75)	
T2/FLAIR hyperintensity	64.0% (73/114)	*retained*	*65.3% (49/75)*
Volume (cm3)	17.83 [6.31 – 35.87]	*co-deleted*	*33.3% (25/75)*
Tumor depth		*1p-retained, 19q-deleted*	*1.3% (1/75)*
*Gyral and subgyral*	*49.1% (56/114)*	MGMT promoter (n=83)	94.3% (83/88)
*Subgyral*	*33.3% (38/114)*	*Methylated*	*56.6 % (47/83)*
*Gyral*	*17.5% (20/114)*	*Unmethylated*	*34.9% (29/83)*
		*Partially methylated*	*8.4% (7/83)*
**Surgical parameters**			
Previous craniotomy			
*No*	*55.3% (63/114)*		
*Yes*	*44.7% (51/114)*		
Anesthesia technique			
*General anesthesia*	*95.6% (109/114)*		
*Awake craniotomy*	*4.4% (5/114)*		
Laterality			
*Left*	*50.0% (57/114)*		
*Right*	*50.0% (57/114)*		
Resection after iMRI	71.1% (81/114)		
Complete ultrasounds	80.7% (92/114)		

## Footnotes

1
https://github.com/ReubenDo/pydeface-niftyreg

2 Available at https://www.slicer.org

## References

[1] Bastos D. C. D. A. Challenges and opportunities of intraoperative 3D ultrasound with neuronavigation in relation to intraoperative MRI. Front. oncology 11, 656519 (2021). 10.3389/fonc.2021.656519 PMC8139191 34026631

[2] Dorward N. L. Postimaging brain distortion: magnitude, correlates, and impact on neuronavigation. J. neurosurgery 88, 656–662 (1998). 10.3171/jns.1998.88.4.0656 9525711

[3] Nabavi A. Serial intraoperative magnetic resonance imaging of brain shift. Neurosurgery 48, 787–798 (2001). 11322439 10.1097/00006123-200104000-00019

[4] Nimsky C. Quantification of, visualization of, and compensation for brain shift using intraoperative magnetic resonance imaging. Neurosurgery 47, 1070–1080 (2000). 11063099 10.1097/00006123-200011000-00008

[5] Orringer D. A., Golby A. & Jolesz F. Neuronavigation in the surgical management of brain tumors: current and future trends. Expert. review medical devices 9, 491–500 (2012). 23116076 10.1586/erd.12.42 PMC3563325

[6] Mercier L. Online database of clinical mr and ultrasound images of brain tumors. Med. physics 39, 3253–3261 (2012). 10.1118/1.4709600 22755708

[7] Rivaz H., Chen S. J.-S. & Collins D. L. Automatic deformable MR-ultrasound registration for image-guided neurosurgery. IEEE transactions on medical imaging 34, 366–380 (2014). 25248177 10.1109/TMI.2014.2354352

[8] Gerard I. J. Towards a second brain images of tumours for evaluation (BITE2) database. In Brainlesion: Glioma, Multiple Sclerosis, Stroke and Traumatic Brain Injuries: Second International Workshop, BrainLes 2016, with the Challenges on BRATS, ISLES and mTOP 2016, Held in Conjunction with MICCAI 2016, Athens, Greece, October 17, 2016, Revised Selected Papers 2, 16–22 (Springer, 2016).

[9] Xiao Y., Fortin M., Unsgård G., Rivaz H. & Reinertsen I. REtroSpective Evaluation of Cerebral Tumors (RESECT): A clinical database of pre-operative MRI and intra-operative ultrasound in low-grade glioma surgeries. Med. physics 44, 3875–3882 (2017). 10.1002/mp.12268 28391601

[10] Behboodi B. RESECT-SEG: Open access annotations of intra-operative brain tumor ultrasound images. arXiv preprint arXiv:2207.07494 (2022).

[11] Ellison D. W. cIMPACT-NOW update 4: diffuse gliomas characterized by MYB, MYBL1, or FGFR1 alterations or BRAF V600E mutation. Acta neuropathologica 137, 683–687 (2019). 30848347 10.1007/s00401-019-01987-0

[12] Ellison D. W. cIMPACT-NOW update 7: advancing the molecular classification of ependymal tumors. Brain Pathol. 30, 863–866 (2020). 32502305 10.1111/bpa.12866 PMC8018155

[13] Louis D. N. cIMPACT-NOW update 6: new entity and diagnostic principle recommendations of the cIMPACT-Utrecht meeting on future CNS tumor classification and grading (2020). 10.1111/bpa.12832 PMC8018152 32307792

[14] Wesseling P. & Capper D. WHO 2016 Classification of gliomas. Neuropathol. applied neurobiology 44, 139–150 (2018). 10.1111/nan.12432 28815663

[15] Tokuda J. OpenIGTLink: an open network protocol for image-guided therapy environment. The Int. J. Med. Robotics Comput. Assist. Surg. 5, 423–434 (2009). 10.1002/rcs.274 PMC2811069 19621334

[16] Modat M. Global image registration using a symmetric block-matching approach. J. medical imaging 1, 024003–024003 (2014). 10.1117/1.JMI.1.2.024003 PMC4478989 26158035

[17] Fedorov A. 3D Slicer as an image computing platform for the Quantitative Imaging Network. Magn. resonance imaging 30, 1323–1341 (2012). 10.1016/j.mri.2012.05.001 PMC3466397 22770690

[18] Clunie D. A. Dicom3tools Software. https://www.dclunie.com/dicom3tools.html (2018).

[19] Herz C. DCMQI: an open source library for standardized communication of quantitative image analysis results using DICOM. Cancer research 77, e87–e90 (2017). 29092948 10.1158/0008-5472.CAN-17-0336 PMC5675033

[20] Yaniv Z., Lowekamp B. C., Johnson H. J. & Beare R. Simpleitk image-analysis notebooks: a collaborative environment for education and reproducible research. J. digital imaging 31, 290–303 (2018). 10.1007/s10278-017-0037-8 PMC5959828 29181613

